# Induction of apoptosis and ferroptosis by a tumor suppressing magnetic field through ROS-mediated DNA damage

**DOI:** 10.18632/aging.102836

**Published:** 2020-02-18

**Authors:** Lin-Qing Yuan, Can Wang, Dong-Fang Lu, Xia-Di Zhao, Lin-Hua Tan, Xi Chen

**Affiliations:** 1 Department of Central Laboratory, The Children’s Hospital, Zhejiang University School of Medicine, National Clinical Research Center for Child Health, Hangzhou, Zhejiang, China

**Keywords:** antitumor treatment, magnetic field, reactive oxygen species, DNA damage, apoptosis, ferroptosis

## Abstract

Magnetic field (MF) is being used in antitumor treatment; however, the underlying biological mechanisms remain unclear. In this study, the potency and mechanism of a previously published tumor suppressing MF exposure protocol were further investigated. This protocol, characterized as a 50 Hz electromagnetic field modulated by static MF with time-average intensity of 5.1 mT, when applied for 2 h daily for over 3 consecutive days, selectively inhibited the growth of a broad spectrum of tumor cell lines including lung cancer, gastric cancer, pancreatic cancer and nephroblastoma. The level of intracellular reactive oxygen species (ROS) increased shortly after field exposure and persisted. Subsequently, pronounced DNA damage and activation of DNA repair pathways were identified both in vitro and in vivo. Furthermore, use of free radical scavenger alleviated DNA damage and partially reduced cell death. Finally, this field was found to inhibit cell proliferation, and simultaneously induced two types of programmed cell death, apoptosis and ferroptosis. In conclusion, this tumor suppressing MF could determine cell fate through ROS-induced DNA damage, inducing oxidative stress and activation of the DNA damage repair pathways, eventually lead to apoptosis and ferroptosis, as well as inhibition of tumor growth.

## INTRODUCTION

Magnetic field (MF) can be categorized to static and dynamic fields. Electromagnetic field (EMF) can be produced by alternating currents (AC). Domestic power cords generate EMF with the frequency of 50–60 Hertz (Hz), which is an extremely low-frequency electromagnetic field (ELF-EMF, 3-3000 Hz). Based on some epidemiology studies focusing on the relationship between the distribution of power cables and the occurrence rate of leukemia [1], WHO International Agency for Research on Cancer classified ELF-EMF as possible carcinogen to humans [2]. On the other hand, the antitumor effect of ELF-EMF and its synergism with conventional chemotherapy is well documented in the literature [3–5]. With the advantage of being non-invasive and low in toxicity, EMF is an ideal alternate antitumor therapeutic option. A medical device based on an EMF of 200 kHz, Optune® (Novocure, Israel), has been approved by USFDA and EU to be used in clinical treatment for relapsed and primary glioblastoma [6]. A frequency-depedent antiproliferative effect of EMF that ranged from 100 Hz to 21 kHz in various cancer cell lines has been reported [7]. Recently, Voyager® (Mulate Therapeutics, USA), a therapeutic device using radio-frequency EMF, has also been proved effective against brain tumors in clinical trials [8, 9]. Similar with ELF-EMF, static magnetic field (SMF) also has both carcinogenic and antitumor potential, and could enhance the efficacy of chemotherapeutic drugs as well as EMF [10]. Application of ELF-EMF, either unmodulated or modulated by SMF, as antitumor therapeutics, is still under preclinical research.

Both EMF and SMF can target a variety of macromolecules including microtubules and ion channels that can potentially mediate the antitumor effect [5, 10]. However, the targets of primary reactions remain unraveled. It has been demonstrated that concentration of free radicals in biological samples are increased after short-term exposure of either static or dynamic MF [11–13]. Having single unpaired electrons in their outer shell, free radicals are highly oxidizing. Meanwhile, they can be neutralized by the intracellular antioxidative defense mechanisms composed of reducing chemicals, macromolecules and enzymes, such as vitamins C and E, glutathione, nicotinamide adenine dinucleotide phosphate (NADPH), superoxide dismutase, etc. In biological systems, reactive oxygen species (ROS) is the dominating category of free radicals. ROS is composed of free oxygen radicals such as superoxide anion (•O_2_^−^) and hydroxyl radical (•OH), and of non-radical ROS such as hydrogen peroxide (H_2_O_2_), and of organic hydroperoxides. The intracellular ROS level is a dynamic balance between constant generation and elimination. ROS in low concentrations can serve as intracellular signaling messengers through modification of protein structure and functions by oxidizing protein thiol groups; while higher levels of ROS can nonspecifically attack biomacromolecules including DNA, lipids and proteins to interfere with multiple cellular events, such as cell cycle progression, cell metabolism, differentiation, motility, survival, et al [14–16], many of them are candidate targets in cancer therapy.

In our previous studies, a 50 Hz ELF-EMF modulated by SMF with a time-averaged intensity of 5.1 militesla (mT), has been shown to inhibit the proliferation of nephroblastoma and neuroblastoma cells, and to sensitize the tumors to conventional chemotherapy [17]. A similar modulated field was previously found to promote apoptosis in breast cancer and colon adenocarcinoma [3, 18]. In this study, the biological targets and mechanisms of this tumor suppressing field have been further investigated, with particular focus on oxidative damage and cell fate determination.

## RESULTS

### MF selectively inhibited malignant tumor cells

**Figure 1:**
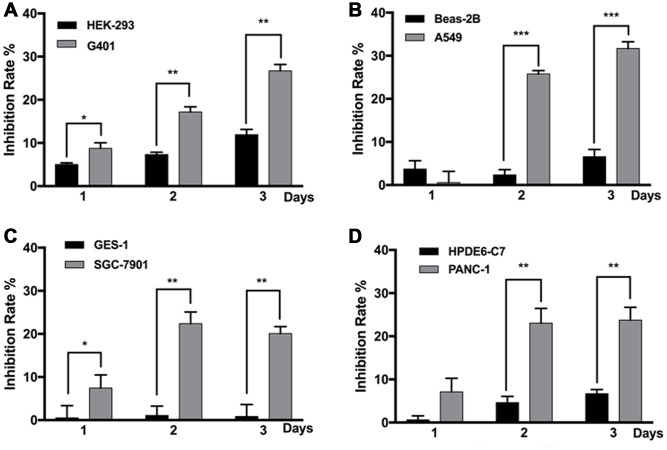
**MF exposure selectively inhibited the growth of various cancer cell lines.** G401 and HEK293 (**A**) A549 and BEAS-2B (**B**) SGC-7901 and GES-1 (**C**) PANC-1 and HPDE6-C7 (**D**) cells were subjected to MF exposure protocol illustrated in Supplementary Table 1, or sham exposure, 2 h daily for 3 consecutive days. Inhibition rate on each day was calculated based on cell viability assays. Results are expressed as mean ± SD (n=5). *: P<0.05; **: P<0.01.

All tumor cell lines used in this study were anaplastic cells derived from highly malignant cases, and non-malignant cell lines derived from the corresponding normal tissues were used in comparison. Most cell lines responded to MF exposure; however, compared with the non-malignant counterparts, the tumor cells were more susceptible to MF-induced inhibition. In Figure 1, on day 1 after 2 h of exposure, inhibition rate in nephroblastoma G401, lung epithelial cancer A549, gastric cancer SGC-7901, and pancreatic cancer PANC-1 was all significantly higher than the corresponding normal cell lines. Inhibition persisted to day 2 and 3, with accumulated MF exposure for 4 and 6 hours respectively. Peak inhibition rates of 20-30% were achieved: 29% in G401 on day 3, 30% in A549 on day 3, 22% in SGC-7901 on day 2, and 24% in PANC-1 on day 3. The most MF-sensitive normal cell line was human embryonic kidney cell HEK293, in which peak inhibition was 12% on day 3 (Figure 1A), while gastric epithelial cell GES-1 remained totally resistant to MF exposure (Figure 1C). It is noted that in the repeated experiments performed in this project, tumor inhibition on exposure day 1 was not always significant, due to the sometime high data deviation. However, same trend of MF-induced inhibition was constantly observed on day 1. Tumor inhibition on exposure day 2 and 3 was always significant since higher inhibition rates had been achieved.

### MF sensitized tumor cells to conventional chemotherapy

**Figure 2:**
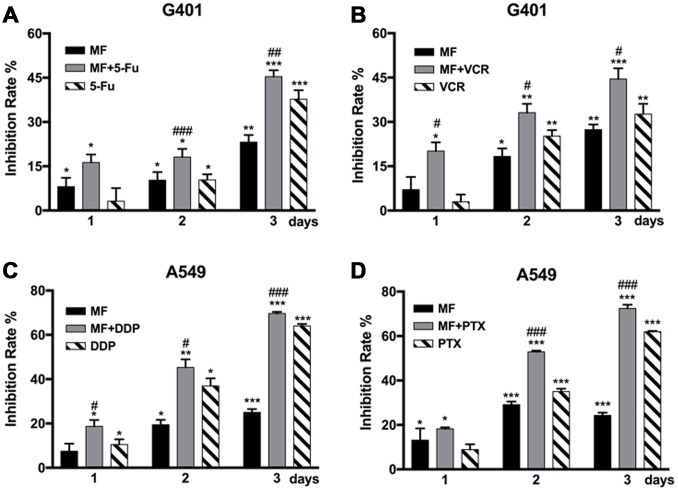
**MF exposure sensitized tumor cells to chemotherapy.** (**A**, **B**) G401 cells were treated by 5-fluorouracil (5-FU, 1.5 μM) or by vincristine (VCR, 2 nM) for 3 days, with or without daily MF exposure for 2 h. (**C**, **D**) A549 cells were treated by cisplatin (DDP, 2.5 μM) or by paclitaxel (PTX, 0.5 nM) for 3 days, with or without daily MF exposure for 2 h. Inhibition rates were calculated based on cell viability assays. Results are expressed as mean ± SD (n=5). *: P<0.05; **: P<0.01; ***: P<0.001, compared with sham exposure group; #: P<0.05; ##: P<0.01; ###: P<0.001, compared with the chemotherapy group.

MF was used in combination with two types of chemotherapeutic drugs, antimetabolites that interfere with synthesis of nucleic acids including 5-fluorouracil (5-FU) and cisplatin (DDP), as well as antimitotic drugs targeting the microtubule including vincristine (VCR) and paclitaxel (PTX). MF exposure could sensitize G401 cells to 5-FU and VCR treatments (Figure 2A, 2B), and to DDP and PTX in A549 cells (Figure 2C, 2D). Significant sensitization effect was observed from either day 1 or day 2, and continued to day 3. Combination of MF with chemotherapy increased cell inhibition rates compared with the sole use of either MF or individual drugs, although no synergy or superimposition effect was observed in these experiments.

### The antitumor effect of MF was mediated through ROS-dependent mechanisms at least in part

**Figure 3:**
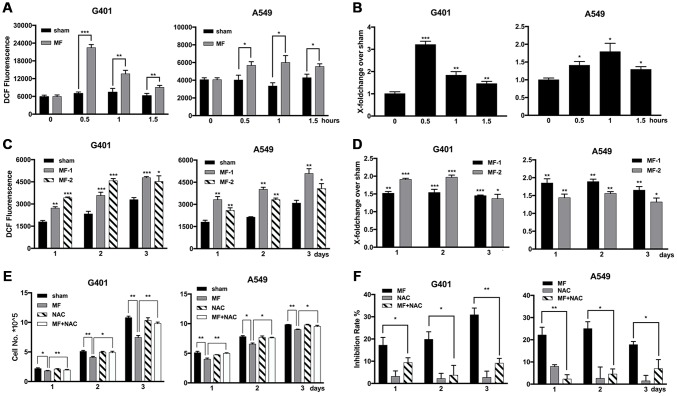
**Elevated levels of ROS induced by MF exposure and use of ROS scavenger to decrease the antitumor effect.** (**A**, **B**) G401 and A549 cells were subjected to MF exposure protocol in Supplementary Table 1, or sham exposure, 2 h daily for 3 consecutive days. ROS levels were measured daily after termination of exposure, shown either in the absolute value of fluorescence emission (**A**), or in fold of change from the MF group over the control group (**B**). (**C**, **D**) In G401 and A549 cells, during the 3-day exposure procedure, ROS levels were measured either right after exposure terminated (group MF-1), or on the next day after termination of exposure for 24 h (group MF-2). ROS levels were indicated either in the absolute value of fluorescence emission (**C**), or in fold of change (**D**). (**E**, **F**) G401 and A549 cells were subjected to the same exposure protocol, with or without incubation with NAC (1 mM). Cell growth curves (**E**) and the calculated inhibition rates (**F**) are presented. Data are expressed as mean ± SD (n=5). *: P<0.05; **: P<0.01; ***: P<0.001.

Elevation of ROS levels was found after short-term MF exposure in both G401 and A549 cells, significant from 30 min, and reached the peak at 30-60 min (Figure 3A, 3B). In G401 cells, ROS level increased about 3 folds in 30 min (Figure 3B). Furthermore, the elevation persisted to the next day, even after exposure had been terminated for 24 h (Figure 3C, 3D). The free radical scavenger N-acetyl-cysteine (NAC, > 0.5 mM) could alleviate but could not eliminate MF-induced ROS production (Supplementary Figure 1). Use of NAC (1 mM) decreased the inhibitory effect of MF in G401 and A549 cells (Figure 3E, 3F), albeit cell growth was not completely restored.

### MF induced intracellular oxidative stress

**Figure 4:**
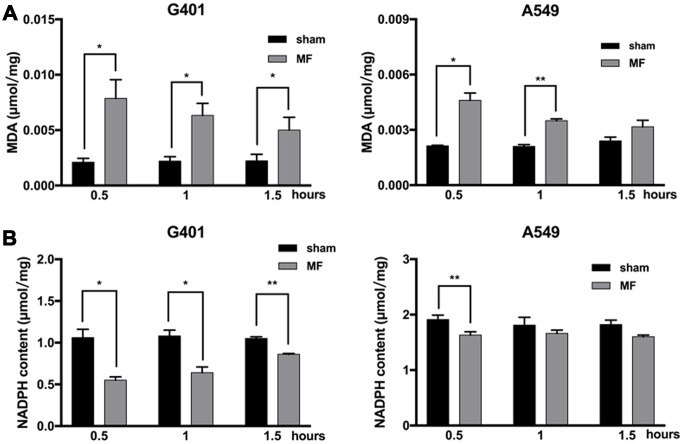
**Oxidative stress following MF exposure.** G401 and A549 cells were subjected to MF exposure for 0.5, 1 or 1.5 h. (**A**) MDA content as indicator of lipid peroxidation. (**B**) NADPH content as indicator of antioxidative capacity. Data are expressed as mean ± SE from 3 independent experiments (n=5 in each experiment). *: P<0.05; **: P<0.01.

Malondialdehyde (MDA), an end product of lipid peroxidation and a common marker for ROS-related cellular injuries, especially for cell membranes, was found to be increased in G401 cells, at 0.5, 1 and 1.5 h after MF exposure, and reaching the peak at 0.5 h as 3.7 times higher than the basal level from the control group. In A549 cells, MDA level increased at 0.5 h and 1 h of exposure, and reaching the peak at 0.5 h as 2.2 times higher than the basal level (Figure 4A). On the other hand, NADPH, a reducing molecule that serves as a major reservoir of the intracellular antioxidant defense, was found to be decreased in both G401 and A549 following exposure (Figure 4B). In G401, NADPH level decreased at 0.5, 1 and 1.5 h, with a reduction of nearly 50% at 0.5 h. In A549 cells, decrease of NADPH was not as pronounced. A significant reduction of about 15% was observed at 0.5 h, and regular level was restored shortly afterwards. In summary, increase of MDA and decrease of NADPH indicated that MF disturbed the cellular redox balance and induced oxidative stress.

### MF exposure led to ROS-dependent DNA damage and subsequent activation of DNA repair pathways

**Figure 5:**
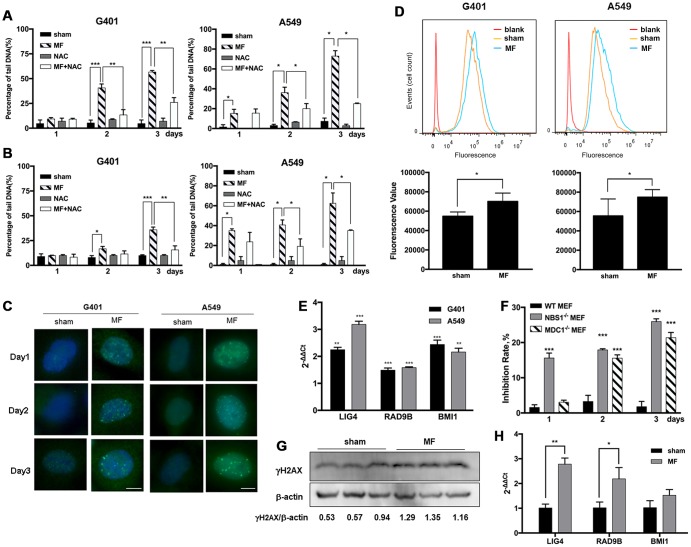
**ROS-induced DNA damage and activation of DNA repair pathways following MF exposure.** G401 and A549 cells were subjected to MF or sham exposure, 2 h daily for 3 consecutive days, with or without incubation with NAC (1 mM). (**A**, **B**) Percentage of tail DNA detected by alkaline (**A**) and neutral (**B**) Comet assays. (**C**) Subcellular localization of γH2AX in G401 and A549 cells. Scale bar= 5 μm. (**D**) Expression of DNA-PKCs protein in G401 and A549 cells with MF exposure (MF) or sham exposure (sham) on day 2, detected by flow cytometry analysis (n=3). *: P<0.05. (**E**) mRNA expression of genes in DNA repair system including *LIG4*, *RAD9B* and *BMI1* in G401 and A549 cells. Asterisk indicates comparison with sham exposure (n=3). (**F**) WT and *MDC1* or *NBS1* deficient MEF cells were subjected to the same MF exposure protocol. Inhibition rates were calculated from number of viable cells. Data are expressed as mean ± SE from 3 independent experiments (n=5 in each experiment). Asterisk indicates comparison with WT-MEF. (**G**, **H**) G401 nephroblastoma was established in nude mice. Expression of γH2AX protein (**G**) and selected genes from the DNA repair system (**H**) are shown (n=3). *: P<0.05; **: P<0.01; ***: P<0.001.

**Figure 6:**
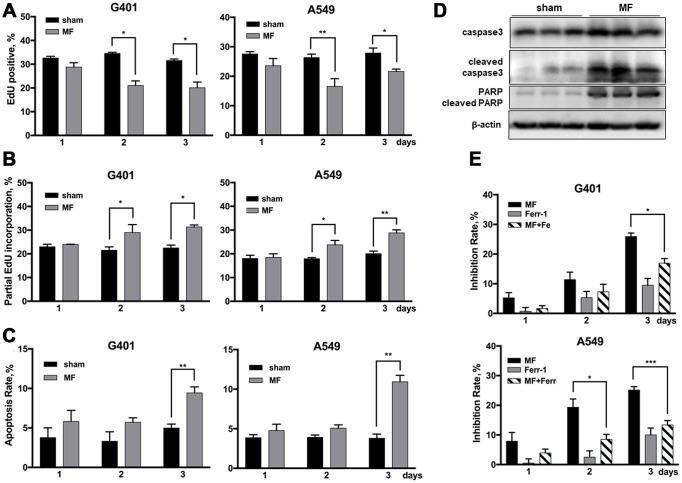
**Cell fate following MF exposure.** G401and A549 cells were subjected to MF or sham exposure, 2 h daily for 3 consecutive days. (**A**, **B**) EdU incorporation assay to detect the ratio of EdU positive nuclei (**A**), and ratio of nuclei with partial EdU incorporation (**B**). (**C**) Cell apoptosis rates measured by flow cytometry. (**D**) Expression of PARP and caspase 3 (precursor and cleaved forms) in G401 nephroblastoma xenografts established in nude mice. (**E**) Ferroptosis detected by co-incubation with ferrostatin-1 (Fer-1, 0.5 μM, 12 h per day) together with MF exposure in G401 and A549. Results are expressed as mean ± SE from 3 independent experiments (n=5 in each experiment). *P < 0.05, **: P<0.01; ***: P<0.001.

Alkaline Comet assay detects both single and double strand breaks (SSB and DSB) of DNA, and neutral Comet assay detects DSB only. In G401 and A549 cells, MF induced pronounced SSB and DSB on both day 2 and day 3, as indicated by increase in percentage of DNA in the tails detected by alkaline and neutral Comet assays (Figure 5A and 5B). On day 3 in G401 cells, statistically more DNA damage was detected by alkaline Comet assay (57% of tail DNA) compared with those detected by neutral Comet assay (36%), suggesting DSB to be the more dominating type of damage. Similar phenomenon was observed in A549 cells, with 73% of the cells with DSB and 62% with SSB. DNA damage was also confirmed by detection of partial incorporation of 5-ethynyl-2'-deoxyuridine (EdU) into the cell nuclei shown in Figure 6B. In both cell lines, from exposure day 2, the amount of cells with pronounced DNA damage and repair was significantly higher compared with sham exposure, and the same on day 3 (Figure 6B, Supplementary Figure 4). Meanwhile, γH2AX, a marker of DNA damage and activation of the homologous recombination DNA DSB repair pathway at the early stage, was recruited to the nuclei of both G401 and A549 cells shortly after exposure (Figure 5C). By flow cytometry analysis, DNA-PKcs, another protein mediating the nonhomologous end- joining DNA DSB repair pathway, was also found to be increased on exposure day 2 in G401 and A549 cells (Figure 5D). Expression levels of several genes involved in the DNA repair pathways, *LIG4, RAD9B* and *BMI1*, were increased in both G401 and A549 cells to different extent (Figure 5E). Furthermore, use of ROS scavenger NAC rescued most of the forementioned DNA damage (Figure 5A, 5B). In Supplementary Figure 2, morphology of nuclei and tail DNA by direct imaging of the gels are presented.

To test the association of the antitumor effect and the integrity of DNA damage repair pathways in the circumstance of MF exposure, MEF cells deficient of key DNA repair genes *NBS1* or *MDC1* were used. Data showed that *NBS1* and *MDC1* deficient MEF cells were far more susceptible to MF exposure compared with wild-type MEF, which remained resistant (Figure 5F, Supplementary Figure 3).

Previously in our lab, G401 nephroblastoma xenograft has been successfully established in nude mice. The tumor was fairly resistant to DDP, and MF sensitized the tumor to DDP treatment [17]. In this study, occurrence of DNA damage and activation of repair has also been confirmed in tumor xenografts. After being subjected to MF exposure in vivo, γH2AX expression was increased in tumor tissues (Figure 5G). Expression of poly ADP-ribose polymerase (PARP), anther marker of DNA repair activation and also an apoptotic marker protein, was elevated (Figure 6D). Furthermore, expression of DNA repair genes *LIG4* and *RAD9B* increased significantly at mRNA level in tumor xenografts (Figure 5H).

### Cell fate following MF exposure

Proliferation rate in G401 and A549 cells, indicated by ratio of EdU positive nuclei, decreased about 10% on exposure day 2, and similar on day 3 (Figure 6A, Supplementary Figure 4). Cell apoptosis rate was significantly increased, but for less than 10% (Figure 6C). MF-induced apoptosis in vivo was also examined in G401 tumor tissues from nude mice. Cell apoptosis markers, cleaved caspase 3 and cleaved PARP, were significantly increased upon exposure (Figure 6D). Induction of ferroptosis was analyzed by use of a specific inhibitor, ferrostatin-1(Fer-1), which partially reduced the antitumor effect induced by MF exposure (Figure 6E). In G401, on exposure day 3, growth inhibition declined from 26% to 17% when Fer-1 was used. Similarly, in A549 cells, Fer-1 reduced the inhibition rate from 25% to 13% on day 3.

## DISCUSSION

In this study, a previously reported tumor suppressing field [17] has been further shown to selectively inhibit several types of cancer cells. Similarly in another study, a 50 Hz field with a flux density of 1 mT induced apoptosis in a transformed human squamous cell carcinoma line, but not in a non-transformed human amniotic fluid cell line [11]. It is noted that ELF-EMF fields are sometimes reported to be tumor promotive. In our experiments with this modulated MF, tumor cell lines differed in their susceptibility to field-induced inhibition, but growth promotion has never been observed. What renders tumor cells susceptibility to MF exposure remains unknown at this stage. Based on the findings from this study, differences in intracellular antioxidant reservoir and activity of DNA damage repair system could contribute to the defense mechanisms against MF-induced injuries, rendering cells differences in susceptibility.

Different types of MF are known to be able to sensitize tumor cells to radiotherapy [19] and to reverse chemoresistance. Such examples include ELF-MF in combination with DDP or 5-FU [20, 21], SMF with doxorubicin [22], and pulsed EMF with daunorubicin [23]. We have previously reported the sensitizing effect of this tumor suppressing MF to DDP treatment in nephroblastoma [17]. In this study, this field is further shown to sensitize tumor cells to both antimetabolite drugs (5-FU, DDP) and antimitotic drugs (VCR and PTX), with different potency in different cell lines, suggesting the field could potentially be applied as adjuvant to conventional chemotherapies. The results also suggested the field might have multiple mechanisms to induce tumor inhibition, inclusive of, though not dependent on, oxidative stress, DNA damage, and mitosis arrest.

The field studied is composed of periodical SMF and ELF-MF modulations with time-averaged intensity only 100 times stronger than the geomagnetic field, too low to cause direct damage of chemical bonds in biomolecules. The subsequent biological effect after field exposure may be mediated by free radicals. In our working model, when being subjected to exposure, intracellular ROS increased and reached peak levels within an hour, and the elevation persisted for at least 24 hours, when a next exposure session would be exercised. Meanwhile, increased oxidative stress has been identified by membrane lipid peroxidation and decreased antioxidant reservoir. Theoretically, concentrations of free radicals can be elevated by both SMF and ELF-MF due to Zeeman effect that split the energy levels in certain molecules [24]. In this study, SMF and ELF-MF could induce ROS production separately (Supplementary Figure 5). This is consistent with literature showing elevated ROS concentrations in biological systems regardless of MF types, including SMF [22, 25] and ELF-EMF [26, 27]. Furthermore, elevation of ROS under the modulated field was higher than that induced by either SMF or ELF-MF alone, but was still lower than the superimposition of the two (Supplementary Figure 5). The intracellular concentration of free radicals is the result of the balance between the production and the scavenging capacity of antioxidants that can also be regulated by MF. This explains the sometimes contradictory findings that MF failed to induce free radical elevation in cells even after 15 days [28], or quick recovery to normal levels after termination of exposure [29], as well as the cell type specific effects [30]. From our data, this tumor suppressing field was able to exhaust the intracellular antioxidant reservoir to cause significant oxidative damage over long term.

Free radicals are highly reactive and always lead to non-specific damage of macromolecules including nucleic acids, proteins and lipids. Hydroxyl ions, one specific type of ROS generated via the Fenton reaction, are very reactive towards DNA. Tumor suppressing MF in this study induced ROS-dependent DNA damage including DSB and SSB. This is consistent with previous studies on 50 Hz fields [31, 32]. It is noted that in the literature, ELF-EMF fields frequently fail to induce DNA damage [33, 34]. The two major DNA repair mechanisms for DSB, the more dominating type of DNA damage in our model, are homologous recombination (HR) and nonhomologous end-joining (NHEJ). During HR, γH2AX is recruited to the loci of DSBs to further recruit the ATM kinase, NBS1 and MDC1 proteins are required for ATM phosphorylation. From our data, increase of γH2AX levels were identified in both cell lines and tumor tissues after MF exposure, and the protein was localized in the nuclei. Increased sensitivity of NBS1 and MDC1 deficient cells to MF exposure further proved activation of HR. Meanwhile, NHEJ pathway activation was indicated by the increased expressions of DNA-PKcs protein that is the catalytic subunit of DNA-PK kinase that rejoins DSB [35], and by the increased transcription of *LIG4* gene that encodes ATP-dependent DNA ligase IV [36]. On the other hand, increase of PARP suggested recognition and repair of DNA SSB through base excision repair (BER) [37]. In addition, the field seemed to be able to induce unconventional DNA repair pathways involving *RAD9B* [38] and *BMI* [39] genes. In our model, although DNA damage was pronounced following MF exposure, the activities of the multiple DNA repair mechanisms remain to be evaluated.

The tumor suppressing field in this study seemed to have stronger anti-proliferation potency than proapoptotic. Optune®, the clinically approved tumor treating field, could inhibit mitosis by directly interfering with microtubule dynamics [40, 41], which is also a well-established target of SMF [13, 42]. Although not tested in this study, cell fate determination under MF exposure can also be related to ROS-independent activation of signaling pathways and transcriptional factors. Cell proliferation can be regulated in response to change of redox status [12]. The downstream signaling pathways of over-produced ROS reported in the literature, such as JNK, AKT and MAPK, are also potential effectors of this tumor suppressing field.

Discovered in 2012 [43], ferroptosis is defined as iron-dependent programmed cell death (PCD) as a result of lipid peroxidation, and can be distinguished from other PCD pathways such as apoptosis, necroptosis, and autophagy. Iron and its derivatives, found in ROS-producing and reducing enzymes, are essential for intracellular balance of ROS [44]. Iron and redox imbalance contribute to development of many diseases including cancer [45]. In our working model, oxidative stress and lipid peroxidation, which are pre-existing conditions to induce ferroptosis, were both manifested. From our data, Fer-1, a potent inhibitor of ferroptosis [43], partially reversed MF-induced proliferation inhibition. Thus induction of ferroptosis contributed to the antitumor mechanism. To date, no literature has previously reported induction of ferroptosis by MF. MF may directly enhance influx of ferrous (Fe^2+^) and ferric (Fe^3+^) ions, as known to increase cellular calcium ion [46]. Interestingly, this is in compliance with the strategy of targeted iron overload to treat cancer [47, 48], amongst many other antitumor strategies targeting ferroptosis being investigated. P53, a key mediator of, ferroptosis in tumors [49], has been frequently found to be up-regulated by ELF-EMF [50, 51]. Apart from p53 and its downstream effectors, induction of endoplasmic reticulum (ER) stress is another potential cross-talk mechanism between apoptosis and ferroptosis [52]. Much remains to be investigated to fully reveal the determination of cell fate with multiple means under MF exposure.

In summary, exposure under tumor suppressing MF could selectively inhibit a broad spectrum of cancers. This field could induce ROS-mediated DNA damage, leading to apoptosis and ferroptosis, as well as proliferation inhibition. Further understanding towards the underlying molecular basis of cell fate determination and differential sensitivity is potentially beneficial to guide individual and precision therapies.

## MATERIALS AND METHODS

### MF exposure facility and protocol

A self-constructed MF exposure facility was used in this study, based on a prototype previously established [3]. The characteristics of the MF generated by this facility were published previously [17]. Briefly, modulated MF was generated by a pair of coils mounted horizontally with their axes lying along the same plane and orthogonally to the ground. The coils were connected with a circuit providing direct current (DC) and 50 Hz alternating current (AC), generating SMF and power frequency EMF respectively. The AC current was obtained from the 50 Hz line using a two voltage variable transformer and the DC current was obtained with a bridge. In a standard MF exposure, 50 Hz EMF and SMF were used in combination as illustrated in Supplementary Table 1.

### Cell lines, culture and exposure protocol

Human nephroblastoma cell line G401, human embryonic renal epithelial cell line HEK293, human lung cancer cell line A549, human bronchial epithelial cell line BEAS-2B, human gastric cancer cell line SGC-7901, human gastric epithelial cell line GES-1, human pancreatic cancer cell line PANC-1, human pancreatic ductal epithelial cell line HPDE6-C7, wild-type (WT) mouse embryonic fibroblast (MEF) cells, *NBS1*^-/-^MEF and *MDC1*^-/-^MEF cells were used in this study. G401, HEK293, A549, BEAS-2B, PANC-1 and MEF were originally from American Tissue Culture Collection (ATCC), and were all purchased from the cell bank of the Chinese Academy of Science, Shanghai, China, except for G401 cell line that was purchased from Cell Source Center, IBMS, CAMS/PUMC, Beijing, China. HPDE6-C7 cell line was established in Ontario Cancer Institute, Canada [53], SGC-7901 cell line was established in Fudan University, Shanghai, China [54], and GES-1 cell line was established in Cancer Hospital of Beijing University, China [55]. *NBS1*^-/-^MEF and *MDC1*^-/-^MEF cells were established in the Institute of Translational Medicine, Zhejiang University [56]. G401 cells were cultured in McCoy5A medium (Jinuo, China); A549, HEK293, BEAS-2B, SGC-7901, HPDE6-C7 and MEF cells were cultured in DMEM medium (Jinuo, China); GES-1 and PANC-1 cells were cultured in RPMI 1640 medium (Jinuo, China), all supplemented with 10% fetal bovine serum (Gibco, USA). Cells were cultured in humidified atmosphere at 37°C with 5% CO_2_. As a standard MF exposure protocol in vitro, the exposure system was placed in an incubator (ThermoFisher, USA) to maintain the cell culture condition continuously. The culture dishes were placed in the middle of the exposure chamber, and subjected the protocol illustrated in Supplementary Table 1 for a total of 2 h per day for 3 consecutive days. Control cells were simultaneously placed in another incubator with sham-exposure, i.e., an identical exposure chamber with the power switched off.

### Chemicals and reagents

All assay kits and antibodies were commercially available and purchased from companies indicated below. Laboratory chemicals including 2’,7’-dichlorofluorescein diacetate (DCF-DA), N-acetyl-cysteine (NAC) [57], trypsin, trypan blue, bovine serum albumin (BSA), and Triton X-100 were purchased from Sigma Aldrich, USA. Sources of other chemicals are indicated in the text. The following chemotherapeutic drugs were used: cisplatin (DDP, Haosen Pharmaceutical, Jiangsu, China), vincristine (VCR, Lingnan Pharmaceutical, Guangdong, China), paclitaxel (PTX, Sangon, Shanghai, China), and 5-fluorouracil (5-FU, Sigma Aldrich, USA).

### Assays for cell viability and apoptosis

Cell viability was determined by cell counting kit-8 (CCK8, Dojindo Laboratories, Japan) based on absorbance at 450 nm using a spectrophotometer (Multiscan MK3, ThermoFisher, USA). Alternatively, number of viable cells was counted after trypan blue staining by an automatic cell counter (Countstar IC1000, Ruiyu Biotech, Shanghai, China). Inhibition rate was calculated based on the following formula: (No. cells from the control group – No. cells from the treatment group)/ No. cells from the control group. Cell apoptosis was analyzed by a commercial kit based on AnnexinV-fluorescein isothiocyanate (FITC) and propidium iodide (PI) staining (BD Biosciences, USA). Cells after desired treatments were collected, fixed and stained, then subjected to flow cytometry (Navios, Beckman Coulter, USA). Data were processed by Flowjo software (v10.0, Tree Star Inc, USA).

### EdU incorporation assay

Cell proliferation, as well as occurrence of DNA damage repair, was examined by incorporation of 5-ethynyl-2'-deoxyuridine (EdU, Solarbio, China) that gets incorporated to the nuclei during DNA synthesis [58]. Briefly, cells were incubated with EdU (20 μM) at 37°C for 2 h, washed, and fixed with 4% paraformaldehyde (PFA, Hushi, Shanghai, China) for 30 min at room temperature (RT), followed by permeation with 0.5% Triton X-100, then incubated with reaction buffer for 30 min at RT. Cell nuclei was stained by Hoechst 33342 (Solarbio, China). The cells were observed and photographed under a fluorescent microscope and companion software (Axio Observer Z1, Zeiss, Germany). The proliferation rate was the calculated ratio of the number of cells with nuclei fully incorporated with EdU against the total number of cell nuclei visualized by Hoechst 33342. Meanwhile, loci of EdU partial incorporation in the nuclei indicate unscheduled DNA synthesis, i.e., occurrence of newly synthesized DNA for damage repair [59], was also counted.

### Measurement of intracellular ROS concentration using DCF-DA probe

The production of ROS was determined by measuring the oxidative conversion of cell-permeable DCF-DA into fluorescent dichlorofluorescein (DCF). Cells were incubated with DCFH-DA (10 μM) diluted by serum-free medium in the dark at 37°C for 45 min prior to termination of treatment. Fluorescent emission of each well at 525 nm with the excitation wavelength of 488 nm was measured by a spectrophotometer (Multiscan MK3, ThermoFisher, USA). The concentration of ROS was expressed either as the absolute value of fluorescent emission, or as fold change in respect to the control group.

### Determination of MDA and NADPH content

Contents of intracellular malondialdehyde (MDA) and nicotinamide adenine dinucleotide phosphate (NADPH) indicate oxidative injury (lipid peroxidation) and antioxidation ability, respectively. Both were quantified by commercial kits (both from Beyotime Biotech, China). For measurement of MDA, cells were harvested by trypsinization and cellular extracts were prepared by prechilled lysis buffer containing thiobarbituric acid (TBA), and centrifuged at 15,000 ×g for 15 min in 4°C to collect the supernatant of cell lysate. The absorbance at 532 nm, indicating the relative level of MDA-TBA adduct formed, was measured on a spectrophotometer (Multiscan MK3, ThermoFisher, USA). For determination of NADPH, cells were harvested by trypsinization and incubated in prechilled NADP^+^/NADPH extracting solution, then centrifuged at 12,000 ×g for 10 min in 4°C to collect the supernatant of cell lysate. After 30 min of heating in water bath at 60°C, NADP ^+^ in the sample decomposed to NADPH that is retained. NADPH converts WST-8 to formazan by reduction. The absorbance at 450 nm indicates the relative amount of formazan, which is proportional to NADPH level of each sample. Finally, total protein concentration from the cell lysate was determined by a bicinchonininc acid (BCA) method, and MDA/NADPH levels in each sample were normalized to per milligram of protein.

### Comet assays for DNA strand breaks

After desired treatments, cell suspension was prepared in PBS, and combined with 10× volume of 0.5% low melting point agarose (Invitrogen, USA), then immediately pipetted onto a slide pretreated with 1% normal melting point agarose, followed by solidification at 4°C for 10 min. For the alkaline Comet assay, the slides were immersed in 4°C prechilled alkaline lysis buffer [pH 10], then placed in an electrophoresis tank filled with ice-cold electrophoretic buffer [pH >13] for 1 h to allow DNA unwinding. For the neutral Comet assay, neutral lysis buffer [pH 8] and neutral DNA unwinding and electrophoresis buffer [pH 8.5] were used. Electrophoresis was performed for 25 min at 25 V and 350 mA. The slides were then washed and stained by SYBR GreenI solution (Solarbio, Beijing, China), observed and photographed by fluorescent microscopy. A total of 100 cells per sample were analyzed by CASP software (v1.1.2, Krzysztof Końca, Poland), with the percentage of DNA in tails was used as the parameter for evaluation of DNA strand breaks.

### Immunofluorescence

After desired treatments, G401 and A549 cultured on glass slides were fixed by 4% PFA for 20 min at RT, permeated by 1% Triton X-100, blocked by 5% BSA, then incubated by primary antibody against γH2AX (ab26350, Sigma Aldrich, USA) and secondary antibody (Alexa Fluor TM 488 conjugated goat anti-mouse IgG, Invitrogen, USA). Nuclei were stained by 4',6-diamidino-2-phenylindole (DAPI, Beyotime Biotech, China). Slides were subjected to fluorescent microscopy (Axio Observer Z1, Zeiss, Germany).

### Quantitative reverse transcription polymerase chain reaction (qRT-PCR)

Total RNA was extracted by UNlQ-10 Column Total RNA Purification Kit (Sangon Biotech, Shanghai, China), and reverse transcripted to cDNA using a commercial kit (Takara, Japan). qRT-PCR was performed (Step One Plus, Applied Biosystems, USA) in three independent experiments with five multiple repeats using self-designed primers listed in Supplementary Table 2 (synthesized by Invitrogen, Shanghai, China).

### Establishment of nephroblastoma xenografts in mice and subsequent analysis

The animal study was approved by the ethical committee of Zhejiang University, China, in 2014. Animals were purchased and housed under standard conditions in the animal center of Zhejiang Chinese Medical University, Hangzhou, Zhejiang, China. Treatment procedure complies with the Public Health Service Policy on Humane Care and Use of Laboratory Animals (Office of Laboratory Animal Welfare, National Institutes of Health, 2015). The method of establishing nephroblastoma xenografts and the protocol of MF exposure in vivo were described in our paper [17] as follows. The experiments were conducted in a room, adjacent to the animal house, which maintained controlled housing conditions generally requested for immune-deficient animals. Each mouse was injected with 5×10^6^ G401 cells in the axilla. The tumor mass could be palpated on day 7, when treatment was started. Mice from MF group were exposed to the same exposure system with the same protocol in Supplementary Table 1, for 80 min each day. The sham-exposed animals from the control group were handled and subjected at the same time, to the same conditions in an identical exposure apparatus powered off. The mice were treated for 15 days and sacrificed three days after the treatments were terminated. Tumor tissues were weighed and sectioned for the subsequent analysis including Western blotting for detecting apoptosis and qRT-PCR for expression of genes involved in DNA damage repair pathways. Three tissue specimens from each group were used.

### Flow cytometry analysis

Cells (G401 and A549) were subjected to MF exposure or sham exposure, 2 h per day for 2 consecutive days, and then harvested and washed with phosphate-buffered saline (PBS). Harvested cells were fixed with 4% PFA for 15 min at RT. After washing with PBS, cells were penetrated with 90% ethanol for 10 min on ice. Then cells were washed twice with cold PBS, then resuspended and incubated by primary antibody against DNA-PKcs (#38168, Cell Signaling Tech, USA) for 1 h in PBS containing 0.5% FBS, followed by incubation with the secondary antibody (Alexa Fluor TM 488 conjugated goat anti-mouse IgG, Invitrogen, USA) in the dark for 30 min, washed twice with PBS and resuspended in 300 μL PBS. Cell suspensions without incubation with antibodies were used as blank controls. The data were collected and analyzed using CytoFLEX S instrument (Beckman Coulter, USA) and Flowjo software (v10.0, Tree Star Inc, USA). Three separate experiments were performed and statistical difference was analyzed by paired Student’s t-test.

### Western blotting (WB)

Sectioned tumor tissues in 10–15 mg of weight were homogenized by Dounce homogenizer in radioimmunoprecipitation assay (RIPA) buffer supplemented with protease inhibitors, then spun at 7500 ×g for 10 min to discard cell nuclei and debris. Protein concentration from the supernatant was determined using BCA method. Sodium dodecyl sulfate-polyacrylamide gel electrophoresis (SDS-PAGE) was performed. Proteins were transferred to polyvinylidene fluoride (PVDF) membranes, which were incubated with primary antibodies against caspase 3 (#9662, Cell Signaling Tech, USA), poly ADP-ribose polymerase (PARP, #9532, Cell Signaling Tech, USA), γH2AX (ab26350, Sigma Aldrich, USA), and β-actin (70-ab008-040, Lianke Bio, China), followed by secondary antibodies conjugated to horse radish peroxidase (HRP, the Jackson Laboratory, USA). Membranes were developed using an enhanced chemoluminescence kit (Biological Industries, Israel). Gray-scale images were captured using the G BOX system (Syngene, USA), and quantitated by densitometry analysis using β-actin as the internal control (Image J, v1.50i, NIH, USA).

### Determination and rescue of ferroptosis

Ferroptosis inhibitor ferrostatin-1 (Fer-1, MCE, USA) at 0.5 μM was used. G401 and A549 cells from the control group was treated with 0.01% DMSO; the ferroptosis rescue group was treated with 0.5 μM Fer-1 for 12 h daily; the MF group received exposure for 2 h daily; and the combinational treatment group was pretreated with 0.5 μM Fer-1 for 10 h and then received co-treatment of 0.5 μM Fer-1 and MF exposure for 2 h. Following the indicated treatments, number of viable cells in each group was counted, and inhibition rates were calculated.

### Statistical analysis

Each experimental condition was repeated in at least three independent experiments, with 5 duplicates for each treatment condition in a single experiment. Data were presented as mean ± SD from one representative set of data selected from multiple repeats, or as mean ± SE based on data from three or more independent experiments. Statistical significance between groups was analyzed by the Holm-Sidak method using Prism software (v7.0, GraphPad Software, USA), and P<0.05 was considered significant, P<0.01 was considered highly significant.

## Supplementary Material

Supplementary Figures

Supplementary Tables
