# Supplementary material for: Induction of apoptosis and ferroptosis by a tumor suppressing magnetic field through ROS-mediated DNA damage
Source: Aging (Albany NY). 2020 Feb 18;12(4):3662–81. doi: 10.18632/aging.102836 (PMC7066880; doi:10.18632/aging.102836)
Supplement: Supplementary Figures [file aging-12-102836-s002..pdf]

## SUPPLEMENTARY FIGURES

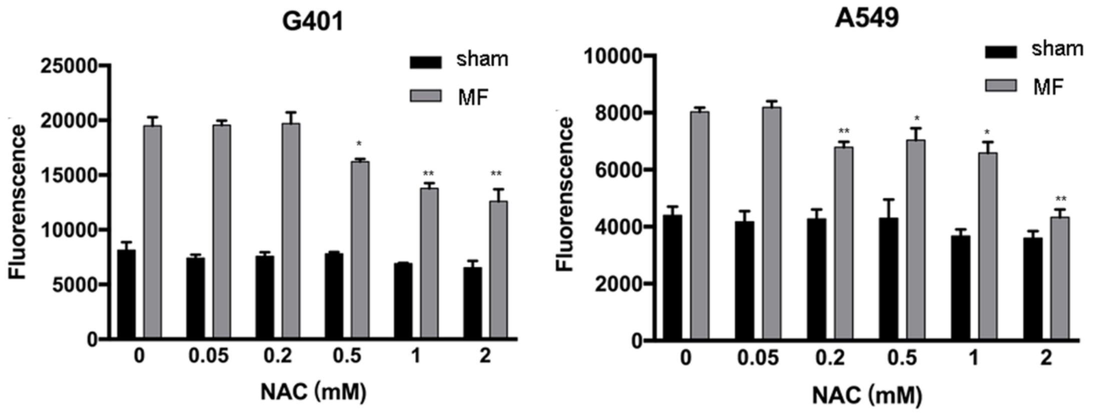


**Supplementary Figure 1. Decrease of intracellular ROS levels by NAC.** G401 and A549 cells were subjected to MF exposure for 0.5 h with or without 2 h' NAC pretreatment at the concentration of 0.05, 0.2, 0.5, 1, or 2 mM. ROS levels were measured by DCFH-DA probe after termination of exposure. Results are expressed as mean ± SD (n=5). *: P<0.05; **: P<0.01, compared with MF group without NAC treatment.

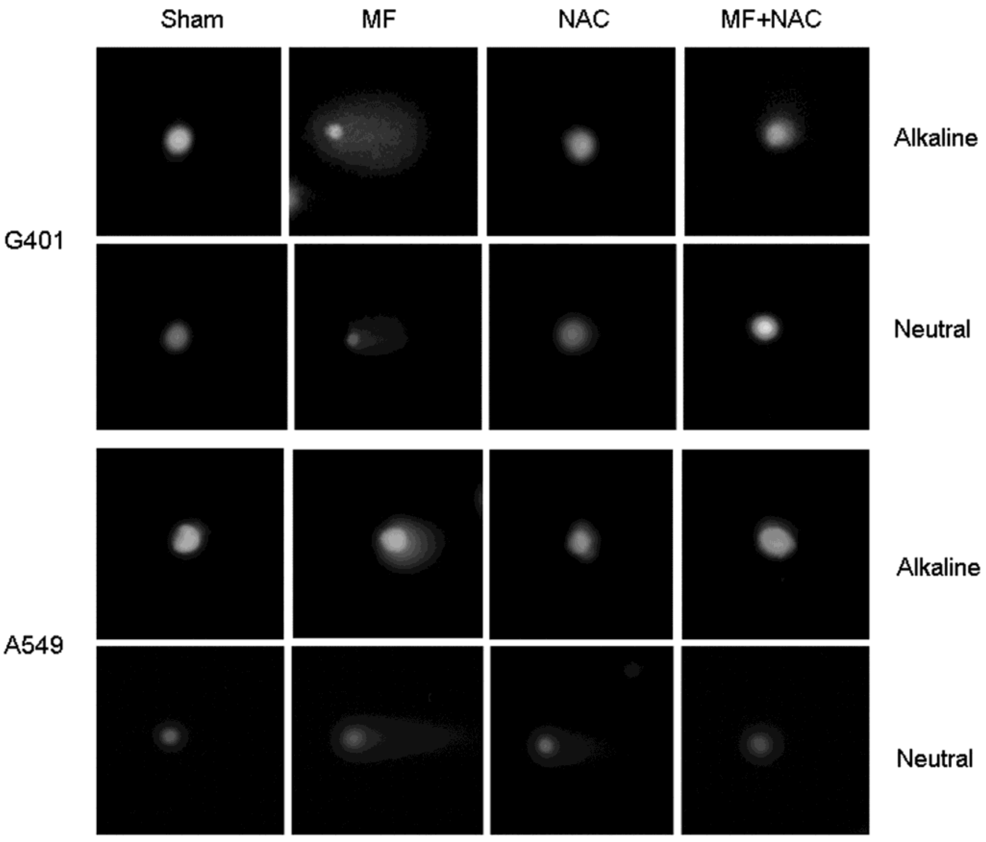


**Supplementary Figure 2. Imaging of DNA tails in the Comet assays.** G401 and A549 cells were subjected to MF exposure for 2 h daily for 3 consecutive days, with or without co-treatment of 1 mM NAC. Representative images of cell nuclei with or without tail DNA on exposure day 2 by alkaline and neutral Comet assays are shown. Statistics analysis is presented in Figure 5A, 5B.

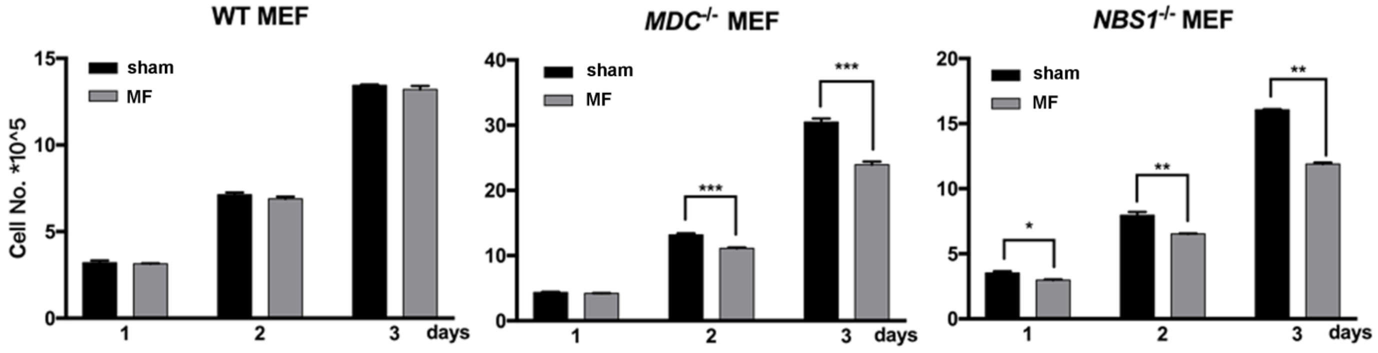


**Supplementary Figure 3. Growth curves of wild-type (WT) and DNA repair deficient MEF cells following MF exposure.** Wild-type (WT), *MDC*$^{-/-}$ and *NBS1*$^{-/-}$ MEF cells were subjected to MF exposure for 2 h daily for 3 consecutive days, or sham exposure. Number of viable cells was counted daily after treatments were terminated. Results are expressed as mean ± SD (n=5). *: P<0.05; **: P<0.01; ***: P<0.001.

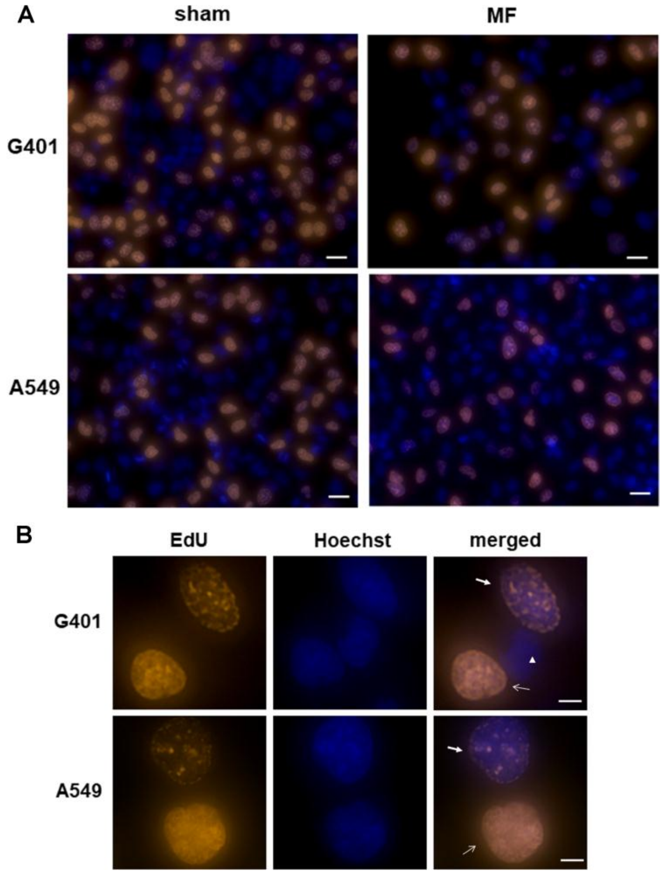


**Supplementary Figure 4. Visualization of EdU incorporation assay.** G401 and A549 cells were subjected to MF or sham exposure for 2 h daily for 3 consecutive days. (**A**) Representative overview of EdU and Hoechst 33342 fluorescence in G401 and A549 cells on exposure day 2. Scale bar= 20 µm. (**B**) Representative images showing EdU positive nuclei (fine arrows) and partial incorporated nuclei (bold arrows). Triangle indicates nuclei without EdU incorporation. Scale bar= 5 µm. Statistics analysis is presented in Figure 6A, 6B.

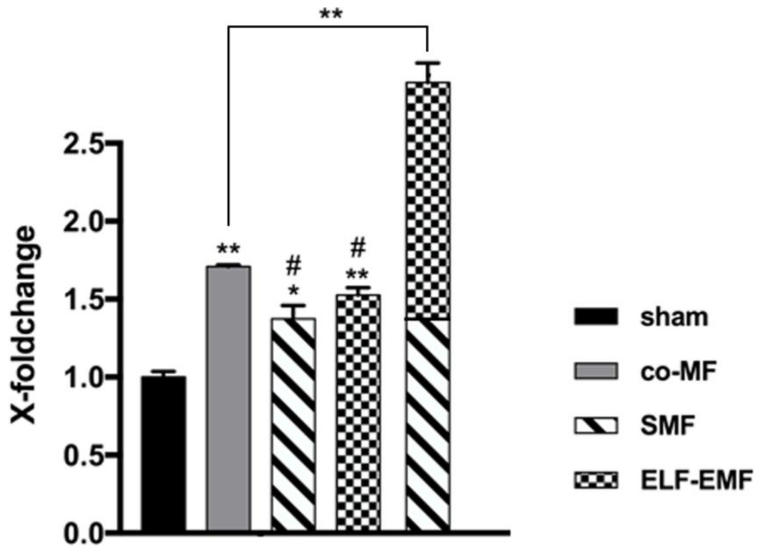


**Supplementary Figure 5. Change of intracellular ROS concentration under exposure of different types of fields.** G401 cells were exposed under modulated MF (Supplementary Table 1, co-MF), under SMF only, under ELF-EMF only, or with sham exposure. ROS was measured after exposure for 1.5 h. Y-axis indicates fold of change compared with the group with sham exposure. Data were shown as mean ± SE from three independent experiments (n=5 in each experiment). *: P<0.05, **: P<0.01, compared with sham, or comparison of the groups indicated; #: P<0.05, ##: P<0.01, compared with co-MF.
