# Supplementary material for: Induction of apoptosis and ferroptosis by a tumor suppressing magnetic field through ROS-mediated DNA damage
Source: Aging (Albany NY). 2020 Feb 18;12(4):3662–81. doi: 10.18632/aging.102836 (PMC7066880; doi:10.18632/aging.102836)
Supplement: Supplementary Tables [file aging-12-102836-s001..pdf]

## SUPPLEMENTARY TABLES

**Supplementary Table 1. Magnetic field exposure protocol.**

| | T1 | T2 | T3 | T4 | T5 | T6 | T7 | T8 |
|---|---|---|---|---|---|---|---|---|
| SMF (mT) | 3 | 4 | 3 | 4 | 3 | 4 | 3 | 4 |
| ELF-EMF (mT) | 1.5 | 2.5 | 1.5 | 2.5 | 1 | 1.5 | 1 | 1.5 |

Extremely low frequency electromagnetic field (ELF-EMF) in 50 Hz are used in combination with static magnetic field (SMF). The field intensities vary periodically, and the time-averaged intensity is 5.1 mT. In a standard exposure protocol in vitro, one exposure is consisted of 4 consecutive sessions, each session composed of 8 rounds with the indicated intensities (T1-8). Each round lasts 3.5 min, making each session 30 min, and one exposure 2 h. Cells are subjected to exposure for 2 h per day for 3 consecutive days. As for in vivo study, the same 8 rounds of fields with varying intensities (T1-8) are exercised, but each round lasts 10 min, making each session a total of 80 min. Mice receive exposure 1 session per day for 15 consecutive days.

**Supplementary Table 2. Sequences of primers used in qRT-PCR.**

| Gene | Primer | Sequence |
|---|---|---|
| *LIG4* | Forward | 5’-TCTCGTTTAACTGCGGCTTGC-3’ |
| | Reverse | 5’-GGGATCTTGGCCTTCCACCC-3’ |
| *RAD9B* | Forward | 5’-GCCCAGCTCAGCCATCATTA-3’ |
| | Reverse | 5’-AACACAGAGGAAGTGCCAGG-3’ |
| *BMI1* | Forward | 5’-AAATCCCGGAAAGAGCAGCC-3’ |
| | Reverse | 5’-CTGGTTGCCCATTGACAGCG-3’ |
| *β-ACTIN* | Forward | 5’-GGACTTCGAGCAAGAGATGG-3’ |
| | Reverse | 5’-AGCACTGTGTTGGCGTACAG-3’ |
